# The Impact of Vegetable Fibres on the Shrinkage and Mechanical Properties of Cob Materials

**DOI:** 10.3390/ma17030736

**Published:** 2024-02-03

**Authors:** Aguerata Kabore, Claudiane M. Ouellet-Plamondon

**Affiliations:** Department of Construction Engineering, Ecole de Technologie Supérieure, 1100 Notre-Dame Street, Montreal, QC H3C 1K3, Canada; claudiane.ouellet-plamondon@etsmtl.ca

**Keywords:** mechanical properties, cob, ductility, volume shrinkage, thermal gravimetric analysis

## Abstract

This study examined the shrinkage rate and mechanical properties of cob samples. Cob is a natural building material composed of clay, water, and varying amounts of plant fibres. The red and beige cob materials in this study containing 3% and 6% wheat fibres were manufactured by hand with clay, bulk fibres (short and long fibres), and a 25% water ratio (water/clay) in order to make their manufacture and use on construction sites feasible and simple. The reference samples were mixed with clay, 25% water, and 0% wheat fibre. The mechanical properties were assessed through compression and flexural tests after 28 and 120 days. The results showed that the fibre addition decreased the bulk density of the composites from 1902 kg/m^3^ to 1264 kg/m^3^. The compressive strength increased from 1.8 MPa to 4.57 MPa for the red clay samples and from 1.65 MPa to 4.61 MPa for the beige clay samples at 28 days. The compressive strength of each mixture decreased slightly with age for the red and beige clay samples, respectively. Conversely, the flexural strength increased with age for the samples reinforced with 3% and 6% fibres. The results also showed that the cob samples can deform without breaking. Increasing the fibre content in the mix resulted in a significant reduction in the shrinkage rate and an increase in the mass loss rate during thermogravimetric analysis. This analysis showed a total mass loss of approximately 5.64%, 6.12%, and 44.87% for the red clay, beige clay, and fibres, respectively. An average volume shrinkage of 1% was observed for the samples with 6% fibre content. The cob discussed in this article can be used as a filling material. In large quantities, it can be made by hand, with feet protected by boots, or with the use of a mixer. The environmental benefits are considerable, as the raw materials are renewable, and the manufacturing process is less energy-intensive.

## 1. Introduction

Earthen construction has recently been attracting increased attention due to its low environmental impact and recyclability. The last decade witnessed growing interest in using ecological and sustainable building materials, driven by the specific requirements for more sustainable and energy-efficient building techniques for the building and construction industry [1]. Clay-based materials bring significant reductions in environmental and climate impacts due to their low carbon footprint [2]. This is also due to their low to near-zero toxicity, high recyclability properties, low transportation costs, and the high energy efficiency and environmental compatibility of the manufacturing processes of various earthen materials [1,3]. Indeed, the construction of one square meter of masonry with compressed earth bricks consumes about 15 times less energy than that of an equal volume of fired bricks [4]. Similarly, a case study on three buildings—a three-story adobe building, a conventional fired clay brick building, and a concrete frame building—showed that the embodied energy of the adobe building was three times less than that of the conventional building and ten times lower than that of the reinforced concrete frame building [4].

Earth is thus being used as a building material in various forms, with recent research focusing on characterizing rammed earth, adobe, and several types of compressed earth blocks [5,6,7,8]. However, few studies have been conducted on the characterization of cob, which is traditionally made from clay soil and bulk fibres mixed with water. Its use as an infill material for wooden structures represents a logical solution because the raw materials involved are clay soil and vegetable fibres, which are abundant and locally available. Several studies have conducted static and dynamic load tests on cob walls to assess their mechanical performance [9,10,11,12] and have found that a compressive strength of 0.6 MPa and a flexural strength of less than 1 MPa are sufficient to provide a safety margin for cob buildings that are at least two stories high, in a non-seismic region [10,13,14]. In addition, cob material and cob walls exhibit good shear behaviour and a long post-peak plastic phase when subjected to flexural tests, which are important characteristics for building materials in seismic zones [10]. Studies on various cob walls have shown that a cob comprising 1.5% straw fibre can withstand high loads with negligible deformation, meaning that buildings constructed with this material can deform without collapsing [15]. Cob buildings can be found in Europe, including Germany, France, and Great Britain; in addition, some are listed as UNESCO World Heritage sites in the World Heritage Earthen Architecture inventory [16]. 

Construction techniques using clay and clay reinforced with plant fibres, such as wood/clay or wood/cob construction, are currently considered sustainable construction practices. However, it is important to note that buildings with significant residual deformations compromise the viability of a durable and resilient city, as well as the lives of its occupants, as their reconstruction after an earthquake requires a lot of time and social resources [17,18]. Therefore, studies have been carried out that show that wood and cob construction extend the lifespan of the building by providing good seismic resilience in its structure [19]. The walls in a wood structure using clay reinforced with plant fibres as a wood-frame filling material are soundproof, termite-resistant, and chemical-free, and the wooden framework offers good seismic resilience in the building structure [17,18,19,20]. Regarding weather resistance, the wooden structure of cob buildings includes a system of diagonal, horizontal, and vertical joints in the corners to be earthquake-resistant [21,22,23]. This diagonal system has flexibility and the necessary strength to support seismic forces during an earthquake. A study conducted on a prototype cob house with a bamboo diagonal system and other studies on wooden and iron systems showed that dynamic tests of the cob model indicated the superior seismic performance of the cob structure [10,24,25,26,27]. A study on the behaviour of pultruded glass fibre-reinforced polymer beam columns for structural building design also demonstrated that these types of beams could be used as lightweight structural components in buildings located in earthquake-prone areas [28]. Due to its lightness and strength, this type of beam can be used in the design of cob-building structures. Before this project study design, an analysis of the measurements of the wooden structure was conducted so that the cob walls would meet the necessary seismic performance for a two-story construction. Despite the fact that studies have been conducted on the combined horizontal and vertical diagonal wooden frame system, a study on diagonally combined systems with a horizontal cob-filled system is necessary to determine the performance of wood/cob walls in seismic zones, given that horizontal systems are considered at risk during long-duration subduction earthquakes [29].

To highlight the mechanical behaviour of vegetable-fibre-reinforced clay materials, several studies have also been conducted on the compressive and flexural strength of different clay mixtures with and without fibres. These studies aim at investigating the use of compressed earth blocks reinforced with plant fibres and consider factors such as fibre length and addition rates [30,31]. Some studies have found that the addition of fibres in compressed earth blocks helps limit crack propagation [30]. Furthermore, it has been observed that the inclusion of cannabis hibiscus fibres with a length of approximately 3 cm and an addition rate of 0.4–0.5% improves both the compressive and flexural strengths. However, beyond these levels, the compressive strength tends to decrease [30,32]. These works highlight the issue of finding the optimal fibre ratio to achieve good mechanical strength. However, in most fibre-reinforced earth construction sites, fibre length measurement is often overlooked, and these fibres are used in bulk for mixing without considering their specific lengths. A study using varying ratios of rice and sugarcane fibres without specifying the length of reinforcing fibres in clay earth materials showed that the increase in the fibre content in the clay earth mixture improved the compressive strength compared to without fibre-reinforced earth materials [33]. According to the study, longer and less porous fibres have a positive effect on the compressive and flexural strengths, enabling the achievement of high compressive strength, even when the mixtures have a large fibre content. In another study, lavender and barley fibres measuring approximately 1 cm in length were used as reinforced fibres in clay materials at percentages of 3% and 6%. The study found that the bricks with 6% fibre exhibited a higher compressive strength than the bricks with 3% fibre [34]. Another author observed a 10 to 20% increase in compressive strength when adding barley fibres measuring 20 mm to 40 mm in length at a percentage of 1.5% [35]. However, for the same fibre length, the compressive strength decreased with the addition of 3 to 5% barley fibre. The reported effect of the plant fibre addition on compressive strength varies from one author to another. Variations in results among different authors can be attributed to differences in the manufacturing techniques used for the test samples, as well as the types and lengths of fibres used. Notwithstanding all these studies, challenges remain when it comes to the full utilization of the potential of cob materials in modern building construction. These challenges include difficulties in reproducing these materials in a real-world setting, taking into consideration the fibre length and the diversity of fabrication techniques of the study samples. In this study, bulk wheat fibres (short fibres + long fibres) were used by adopting the traditional cob-making technique for all mixtures. The length of the fibres was not considered in order to allow the easy reproduction of the two materials obtained in large quantities for construction at building sites. The selection of red and beige clays was based on their widespread use in earthen construction across Africa and other continents. This choice aims to ensure the replication of the materials obtained. The cob mix was manually prepared by hand, with a shovel, and by foot, with the option to use a concrete mixer for large-scale mixing. The manual method was used to ensure that reproduction on different construction sites in Africa can be realized without constraints. The wheat fibres added to the mixture in this study were chosen based on their availability. The tests focused on heavy cob mixes intended for filling the structure of timber-framed buildings, following local construction practices. However, further studies should be conducted by formulating cob mixes with different types of fibres (long + short), using the same sample formulation method to ensure that most plant fibres from various African countries and other continents can be used in the production of cob material under real conditions. The purpose of this study was to investigate the mechanical strength of specimens in cob and clay, the volumetric shrinkage during curing, and post-cracking performance when the specimens were subjected to four-point compression and bending tests at low speeds. The mechanical properties presented in this paper are the average compressive and flexural strength of three replicates of the samples made with 0%, 3%, and 6% wheat fibre (samples left in ambient air for 28 days and 120 days) and the evolution in the volume shrinkage with the drying time.

The studies conducted as part of the present work on the mechanical resistance of the cob are meant to encourage the use of cob as a more environmentally friendly alternative to modern construction materials and to include it among bio-based and geo-sourced materials. Bio-sourced materials come from plants, animals, or microorganisms [36], while geo-sourced materials come from the Earth, such as stone, clay, sand, and other materials obtained from the Earth’s crust [37]. Understanding how cob reacts to mechanical stresses such as compression and flexion makes it possible to integrate cob optimally in the design and construction of buildings. Mechanical resistance tests on the cob provide scientific data on the limits of the loads it can withstand while maintaining structural integrity, as well as a simpler, more reproducible method. These studies would contribute to its recognition as a legitimate construction material that can be used in various applications, such as houses, public buildings, and ecological and sustainable structures. The results of the tests could then also serve as a source of inspiration for innovation in construction techniques and enrich engineering and architecture training programs by providing practical knowledge about traditional materials and their modern applications. Therefore, mechanical resistance tests play a key role in the development and innovation of sustainable construction practices while supporting heritage preservation and advancing scientific knowledge in the field of cob construction. This should position cob as a reliable and viable construction material for hybrid construction. The wood and cob construction would combine the advantages of modern and traditional construction methods. On the one hand, wood construction is favoured for its mechanical performance and economic efficiency; on the other hand, cob construction reinforced with plant fibres is recognized for its ecological and bioclimatic qualities, as indicated in the work of Belabid et al. [38]. 

## 2. Materials and Methods

### 2.1. Clay and Fibre Properties

The two types of clay and the wheat fibre used for the test samples came from Alberta and Quebec, respectively [39]. The density of the two clays was determined by means of the pycnometer method. For this, a 200 g mass of each clay was taken directly from bags and dried in an oven for 24 h at 110 °C. After drying, each clay quantity collected was separated into four 50 g parts after drying, and three parts of each type of clay were used for testing [40]. Tests were carried out following the ASTM D854 guidelines [41]. 

To determine the fibre absorption coefficient, tests were carried out following ASTM D2654 guidelines [42]. The value of the water absorption coefficient, obtained after immersing the fibres in distilled water for 25 min, was used as the water-to-fibre ratio for wetting the fibres before manufacturing the test samples [40]. 

### 2.2. Mineralogical Composition

The mineralogical composition of the clays was determined by X-ray diffraction (XRD), and their chemical composition was analyzed by X-ray fluorescence (XRF) on powdered samples (0–63 µm) selected directly from the clay bags. For the tests, 5 g of each sample was prepared with 25 mL of isopropanol using the XRD mill McCrone apparatus (McCrone group, Westmont, CA, USA). The tests were performed with the Bruker D8 Advance apparatus (Bruker Corporation, Billerica, MA, USA), operating at 40 kV and 40 mA with a Cu source. The tests were performed to identify the main crystalline phases. XRD patterns were measured from 5 to 70° 2 θ, with a step size of 0.020° 2 θ, at a scan speed of 15 tr/min. Crystalline phases were identified by analyzing the peak positions. In X-ray diffraction (XRD), 2θ refers to the angle of diffraction. When X-rays are diffracted by a crystal lattice, the resulting diffraction pattern is observed at an angle of 2θ [43,44]. This angle is used to determine the spacing between crystal lattice planes and is a key parameter in XRD analysis [45].

### 2.3. Thermogravimetric Analysis (TGA/DTG)

A thermogravimetric analysis (TGA) was performed using a Perkin Elmer STA8000 instrument (PerkinElmer. Inc., Billerica, MA, USA). The objective was to determine the weight loss and endothermic reactions of the clay and fibres used in the fabrication of the test samples. The clay samples used for the tests had an average mass of 10 mm to 15 mg and were subjected to heating between 30 °C and 1000 °C at a heating rate of 10 °C/min under an inert gas (nitrogen) of 50 mL/min. The fibres were also subjected to this test.

### 2.4. Production of Test Samples

**Figure 1:**
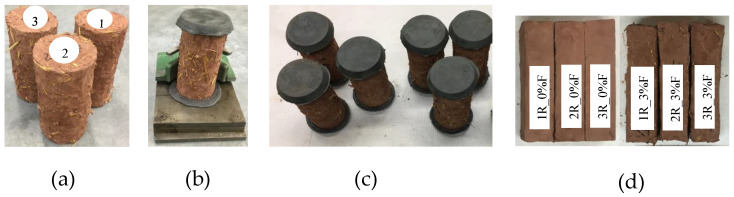
Photographic images of (**a**) the compressive strength samples before surface smoothing, (**b**,**c**) the samples ready for testing, and (**d**) the flexural strength samples.

The procedure for mixing the clays, water, and fibres (0%, 3%, and 6% by mass of fibres) to manufacture the specimens was carried out using the cob manufacturing technique [46]. First, we mixed clay and 25% water (water/clay ratio) before adding moistened wheat fibres at 205% water (water/fibre ratio). Before filling the frames, there was a resting period of 2 h to 3 h after the clay–water–fibre mixture was made. These samples were immediately demoulded and placed in an oven for 10 to 11 h at a temperature of 30 °C. After 10 to 11 h, they were removed from the oven, and drying continued in ambient air (23 °C) until the day of testing. Cylindrical specimens 150 mm in height and 76 mm in diameter were made for the compression tests, and rectangular specimens measuring 150 mm × 38 mm × 38 mm were made for the bending tests. Table 1 presents the proportions of the mix composition for the fabrication of the samples. Additionally, it is worth noting that all the mixtures had the same water/clay and water/fibre ratio. The steps of the procedure for mixing the components of the clay cob samples, including the duration of each step, are presented in Table 2. Figure 1 shows photographic images of (a) the compressive strength samples before surface smoothing, (b) and (c) the samples ready for testing, and (d) the flexural strength samples.

### 2.5. Volume Shrinkage Measurements

The volume shrinkage ($R_{v}$) caused by drying, which is the dimensional change from the wet to the dry state of the material, is calculated by Equation (1) as a function of the drying time, following the ASTM C157 standard guidelines [47], where $V_{0}$ is the initial volume, and $V_{f}$ is the volume after drying:

(1) $$R_{v}=\frac{V_{0}-V_{f}}{V_{0}}\times 100$$

### 2.6. Mechanical Measurements

The tests were conducted on samples aged for 28 days and for 4 months, respectively, to observe the influence of aging on the mechanical properties. Compressive strength tests were performed following the ASTM C39/C39M standard [48], with a load rate of 0.5 kN/s, using a Matest uniaxial compression tester requiring smooth surface samples. Therefore, all samples were first smoothed by placing 145 ℃ molten sulphur mortar on both surfaces that would be in contact with the tester (see Figure 1b). The compressive strength was then calculated using Equation (2), knowing the maximum load at the end of the test and the diameter of the sample:

(2) $$f_{cm}=\frac{4000\;\times F_{max}}{\pi \;\times D^{2}}$$

where $f_{cm}$ is the compressive strength in MPa, *D* is the diameter in mm, and ${\;F}_{max}$ is the maximum load at the break in kN. 

Flexural tests were performed following the standard ASTM C78/C78M [49] using a manual four-point flexural strength tester (ME-8236 (Ayva Educational Solutions, Oakville, ON, Canada)) of a 50 kN load cell. For bending tests, the samples did not require smooth surfaces (Figure 1d). The principle of the test with this apparatus is to determine the maximum load of the failure of specimens placed on two upper supports and two lower supports, with force applied to the middle of the two upper supports under a constant displacement speed of 1 mm/min on average. The distance between the two upper supports was 30 mm, and the distance between the lower supports was 90 mm. Equation (3) was used to calculate the flexural strength after failure:

(3) $$f_{cf}=\frac{F_{max}\times L}{b\;\times {\;d}^{2}}\;\;\;$$

where $f_{cf}$ is the flexural strength in MPa, *L* is the span length in mm, *b* is the nominal width of the samples in mm, and *d* is the depth (thickness in mm). An ANOVA and a *t*-test were conducted on the mechanical strength results to determine the significant and non-significant differences in the results based on the fibre content and the sample age.

## 3. Results

### 3.1. Physical, Mineralogical, and Chemical Characterization

**Figure 2:**
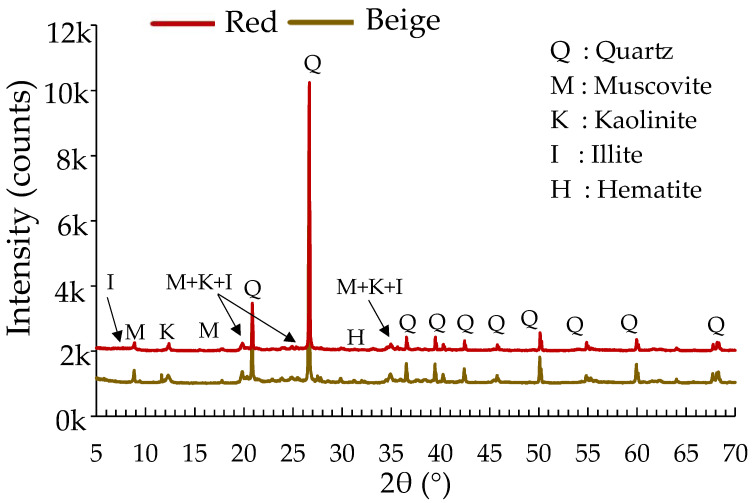
X-ray spectra of the raw clays.

The density obtained after the tests was 2790 kg/m^3^ for the red clay and 2722 kg/m^3^ for the beige clay. For the initial water content, the red clay and the beige clay had initial values of 1.68% and 1.12%, respectively. The density of the clays was used to calculate the total quantity of clays required to produce the test samples. The mineralogical characterization results of the clays by X-ray diffraction (XRD) are presented in Table 3 and Figure 2. The two clays are distinguished by their mineralogical composition, with the common dominant component being quartz, at 43%. The main minerals of the red clay are quartz, muscovite, illite, and small quantities of kaolinite and hematite. For the beige clay, quartz, kaolinite, muscovite, and illite are the main minerals, and hematite is the only trace element. The presence of these mineralogical elements in both clays suggests felsic sources [50]. Two major quartz peaks were observed for 2θ between 20° and 30°. These high peaks indicate the presence of free silica from clay minerals [51]. Quartz is always the most dominant element in the mineralogical composition of clays used in construction [30,52,53].

X-ray fluorescence analysis (XRF) showed that all the tested clays contained mainly silicon dioxide (SiO_2_) and alumina (Al_2_O_3_), with minor impurities of Fe_2_O_3_, K_2_O, and MgO (Table 4). The high SiO_2_ content in the chemical composition of the clays confirmed the high percentage of quartz revealed by XRD analysis [54,55]. The presence of alumina (Al_2_O_3_) is related to the presence of aluminosilicates and kaolinite, while that of iron oxide (Fe_2_O_3_) and potassium (K_2_O) indicates the presence of hematite and illite [51,54,56]. Clays with Fe_2_O_3_ contents below 8% are reported as acceptable clays for the production of building materials [51]. The two clays studied had Fe_2_O_3_ contents of 2.2% and 0.1%, respectively. The addition of fibres to the clay samples did not influence their chemical composition. 

### 3.2. Thermal Thermogravimetric Analysis (TGA) and Differential Thermogravimetric Analysis (DTG)

**Figure 3:**
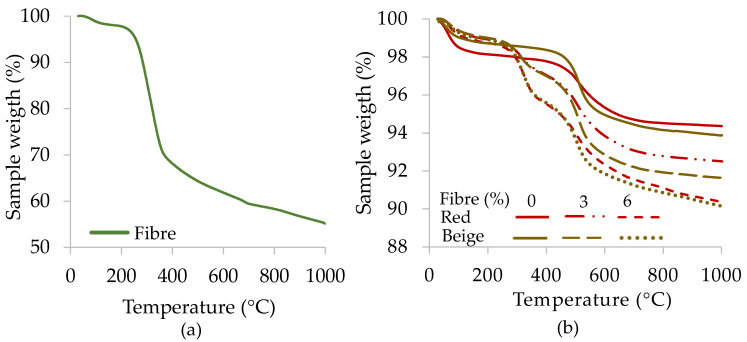
TGA curves: (**a**) fibre sample alone and (**b**) red and beige clay samples without and with fibres.

**Figure 4:**
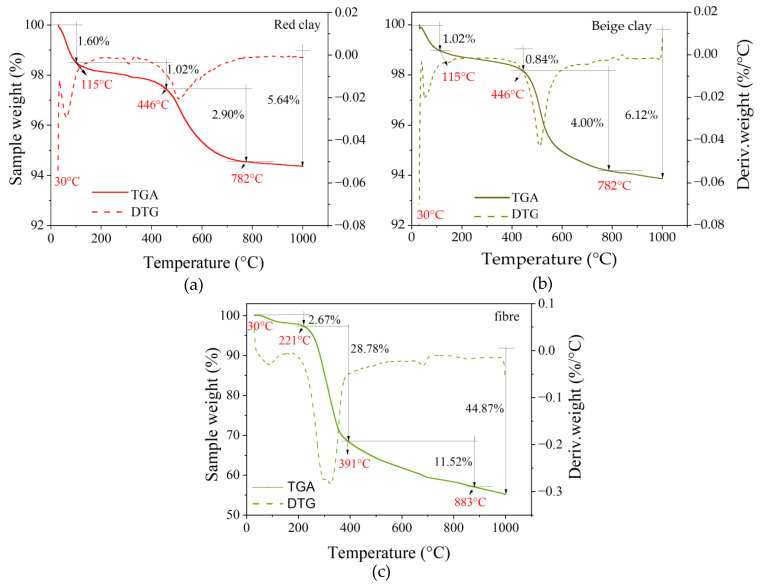
TGA/DTG curves: (**a**) red clay, (**b**) beige clay, and (**c**) wheat fibres.

Figure 3 and Figure 4 present the thermogravimetric analysis (TGA) and differential thermogravimetric analysis (DTG) results for the clay samples with and without wheat fibres. The addition of fibres led to an increase in the mass loss of those samples (Figure 3b).

The TGA-DTG curves in Figure 4 show the endothermic reactions, with the first occurring between 30 °C and 115 °C for the samples of both clays. This first reaction represents the evaporation of water and the disintegration of the unburned volatiles present in the samples [57,58,59,60]. For the red and beige clay samples, mass losses of 1.60 wt% and 1.02 wt% were observed, respectively. The fibres had a mass loss of 2.67 wt%. The second endothermic reaction occurs between 115 °C and 446 °C for the clay samples and between 221 °C and 391 °C for the wheat fibres (Figure 4). This second endothermic reaction showed mass losses of 1.02% and 0.84% for the red clay and beige clay, respectively, and 28.78 wt % for the fibres. This second reaction characterizes the dehydroxylation of goethite for the clay samples [61]. For the wheat fibres, this second endothermic reaction characterizes the decomposition of hemicellulose and cellulose [60,62], with a significant peak at 320 °C.

The third endothermic reaction occurs between 446 °C and 782 °C for the clay samples (Figure 4a,b). The endothermic reactions occurring above 782 °C characterize the dehydroxylation of illite and quartz, followed by a decarbonization reaction due to the decomposition of calcite, resulting in a loss of carbon dioxide [30,61,63,64]. For wheat fibres, the third endothermic reaction occurs between 391 °C and 883 °C, characterizing the degradation of lignin [60]. The maximum decomposition temperature of the wheat fibres was measured at 391 °C. Several authors have reported maximum decomposition temperatures of various plant fibres, including 344 °C for *Althaea officinalis*, 359 °C for okra fibres, 365 °C for jute fibres, 340 °C for sisal fibres, 345 °C for flax fibres, 363 °C for curaua fibres [59], 384 °C for coconut coir fibres, 296 °C for banana fibres, 320 °C pineapple fibres, and 357 °C for sugarcane bagasse fibres [60]. 

### 3.3. Shrinkage Rates of Samples

**Figure 5:**
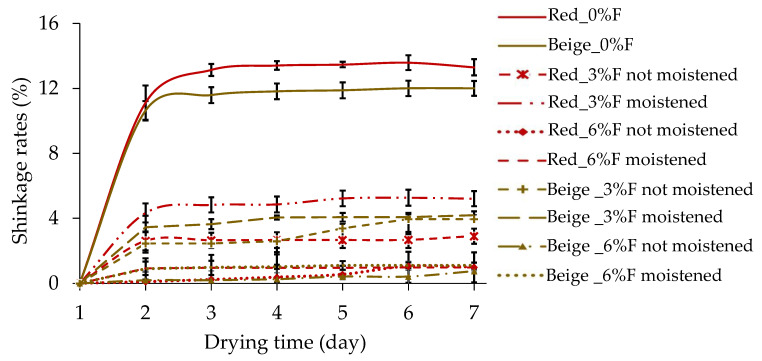
Shrinkage rates versus drying time.

Figure 5 shows the shrinkage rates of the clay samples. We note that the shrinkage evolved with the drying time. As the fibre content increased, less shrinkage occurred. After 7 days of drying, an accumulated shrinkage of 13% was observed for the red clay samples without fibres and of 12% for the beige clay samples without fibres. Depending on the nature and content of the clay, the volume shrinkage during the drying of a clay material should be between 4% and 20% [65]. The shrinkage obtained with the samples of both types of clays was between 4% and 20%. For the samples reinforced with 3% fibre, the shrinkage rate varied between 2% and 5.3% and was a function of whether the fibres used for the mix were wet (Figure 5). For 6% fibre in the mix, the maximum shrinkage rate observed was 1%, irrespective of whether the fibres were wet. The results obtained in this study are consistent with previous findings on samples reinforced with 3%, 5%, and 7% of coir and straw fibres [15]. An increase in the average volumetric shrinkage was observed from the fifth day for samples reinforced with unmoistened wheat fibres. This may be due to the increase in ambient temperature in the laboratory where the samples were stored for drying. It is important to note that the sample storage area is not a controlled space in order to mimic the material’s replication on construction sites, which could result in fluctuations in the ambient climate, leading to slow or rapid drying. Additionally, it should be noted that the moistened and not moistened fibre samples were not produced simultaneously.

### 3.4. Mechanical Properties

#### 3.4.1. Compressive Strength

**Figure 6:**
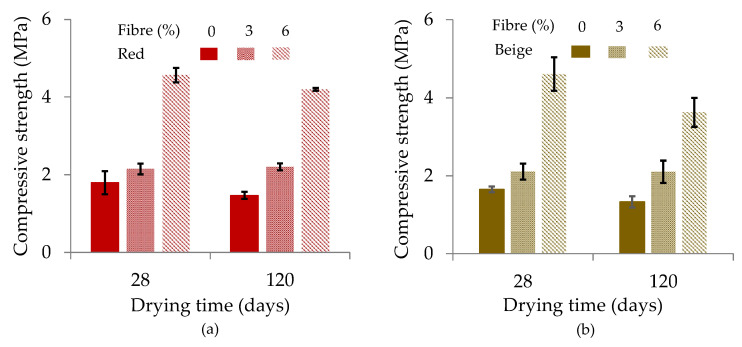
Compressive strengths as a function of drying days: (**a**) red clay samples and (**b**) beige clay samples.

Figure 6 shows the average compressive strengths of three samples per mix and the standard deviations as a function of the number of days of drying (28 days and 120 days of drying). When force was applied for compression testing, the cob material compressed before weakening, unlike the clay samples without fibrous reinforcement, which broke immediately after force was applied (Figure S1 in the Supplementary Materials). In terms of average values, the compressive strength of the red clay samples increased by 20% (reinforced with 3% fibre) and by 154% (reinforced with 6% fibre), and that of the beige clay increased by 28% for the reinforcement with 3% fibre and by 179% for the reinforcement with 6% fibre versus the samples without fibres, respectively (28 days). For the samples dried for 4 months, the increase in the strength was 50% and 186% for the red clay samples reinforced with 3% and 6% fibres, respectively, and the compressive strength of the beige clay samples increased by 58% and 173% for the reinforcement with 3% and 6% fibres, respectively, compared to samples without fibre (Table S1 in the Supplementary Materials). Comparing the results of the samples according to the two drying times shows a slight decrease for all samples, except for the red clay reinforced with 3% fibre, which increased by 3% (Table S2 in the Supplementary Materials). The decrease in the compressive strength after 120 days of drying could be due to the fact that the samples were exposed to ambient conditions with variations in humidity and temperature, resulting in variations in compressive strength. The study of the influence of the water content on the compressive strength was not taken into account in this study and requires further investigation. For the condition of the samples after compression, no breakage of the samples with 3% and 6% fibre was observed, but a detachment of the clay continued to occur from the fibres and the compression (Figure S1b,c in the Supplementary Materials). This can be explained by the presence of fibres. According to previous research, a compressive strength of 0.6 MPa was identified as sufficient to ensure a safety margin for cob buildings at least two stories high [10,13,14]. In the case of this study, the cob samples attained a compressive strength between 2.15 MPa and 4.57 MPa.

#### 3.4.2. Flexural Strength

**Figure 7:**
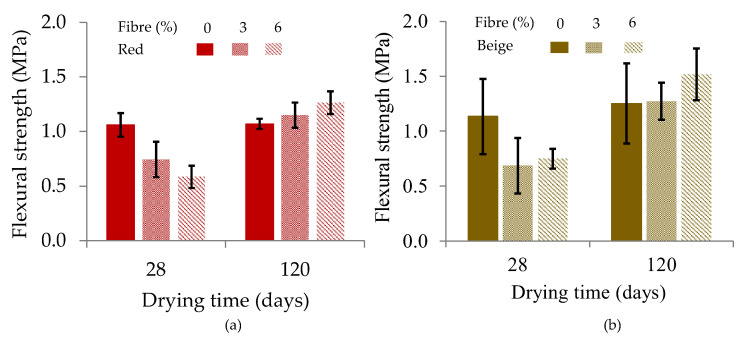
Flexural strengths as a function of the fibre content: (**a**) red clay samples and (**b**) beige clay samples.

Figure 7 shows the changes in the average flexural strength with the drying days (28 days and 120 days). These results show that the flexural strength of all samples without and with fibres increased significantly with the sample age (Table S2 in the Supplementary Materials). This finding is consistent with previous studies [66,67], which support the observed increase in the flexural strength of samples over time. For samples dried for 28 days, the flexural strength decreased with the increasing fibre content in the mixtures (Table S3A in the Supplementary Materials). This decrease may be due to the higher water content in the samples dried at 28 days than those at 120 days. The higher water content in test specimens can lead to a reduction in their flexural strength [68]. After 120 days of drying, the flexural strength of the fibre-reinforced samples increased significantly. The flexural strength values of these samples gradually increased to match those of the samples without fibres (Table S3B in the Supplementary Materials). According to some authors, the decrease in moisture over time in earthen materials is proportional to the increase in their mechanical strength [66,69]. From a mechanical perspective, the results show that wheat fibres influence the flexural and compressive strengths. These results are in agreement with results from several authors who have shown that the length of fibres influences the mechanical strength of the building materials and increases with an increase in the fibre content [31,33,70,71,72]. 

Plant-fibre-reinforced earth materials have a very particular bending failure behaviour. The addition of loose wheat fibres in a clay earth mixture changes the behaviour of the samples without fibres from brittle to semi-ductile deformation and significantly increases the ductility of the materials (Figure S2 in the Supplementary Materials) [73]. As the fibre content increases, the cob materials become more ductile, characterized by plastic behaviour (Figure S2b,c). The results show that the forces applied to the samples with fibres during the tests first propagate into the clay matrix in the form of a crack before being damped by the fibres. This observation was also found during the compressive strength tests (Figure S1 in the Supplementary Materials). Therefore, the clay is used as a binder between the fibres, ensuring the flexibility of the cob materials. Several studies have also shown that earth materials that are not reinforced with fibres experience a quasi-fragile failure when they are in contact with a force. These materials belong to the family of materials with elastoplastic behaviour with brittle failure, such as compressed earth blocks, adobe, cement-stabilized earth blocks, etc. [74]. In this work, the results show that reinforcing the clay material with fibres of different lengths (short and long) improves both the mechanical strength and flexibility of the samples.

### 3.5. The Use of the Studied Cob in Construction

Cob is a material made from clay, straw, and water. It is a filler material for the wooden structure, not a load-bearing material. As the cob has a high density (1200–1700 kg/m^3^), it is very heavy and consistent, so it is necessary to avoid using too much water in the mix. To obtain the kind of heavy cob used in this study, mixing should be carried out with a water/clay ratio of 25% by mass while moistening the fibres with a water/fibre ratio of 205% by mass. The quantity of fibre to be used depends on the quantity of clay (either 3% or 6% by mass of clay). The manufacturing of cob is performed in four steps. For large quantities, these steps are as follows:

- To moisten the fibres, spread them out on a tarpaulin on the ground and dampen the whole (205% W/F), leaving it until the clay/water mixture is ready.
- To mix clay and water, spread the clay on a tarpaulin or in the mixer, pour the required amount of water onto the surface of the clay (25% W/C), and mix until homogeneous.
- For mixes without a mixer, use shovels to bring all the cob together and remix to obtain a homogeneous consistency.
- At the end of mixing, leave to stand for half a day, then spread the wet fibres over the surface of the clay/water mixture and remix to obtain a homogeneous mixture, a consistency that can be used to fill the wooden frame.

After mixing, it is important to obtain a density close to that obtained in the laboratory, between 1200 and 1700 kg/m^3^. The purpose of wetting the fibres before mixing is to optimize the amount of water to be used for cob production, the resting time before use for filling the wooden framework, and to limit the pre-cracking of the cob due to the absorption of moisture into the mix by the fibres. As the fibres are wetted before use, the final mix can be used immediately. Cob walls are designed using two materials with high heat and moisture storage capacities: wood and cob [65]. Before starting to lay the cob, it is important to ensure that the laths or wood framing have a moisture content that allows the cob to adhere easily to the wooden structure. This prevents the wood from drying out too quickly and absorbing moisture from the cob. The cob, a mixture of clay, fibres, and water, is laid in successive layers until the desired thickness is reached, filling the wooden frame from bottom to top. This method was used to produce the test samples in the laboratory.

A raised foundation to protect the structure against capillary rise and collapse due to the building’s load distribution is recommended. The roof must be able to protect the walls against water from driving rain, and a drainage system must be established to prevent water damage.

For the finish, a plaster made of clay, lime, or clay mixed with lime is generally applied to the mud wall and can, therefore, be applied to both exterior and interior cob walls. Indeed, this article aims to evaluate the mechanical properties of cob material manufactured using the traditional method, using current characterization methods such as ASTM C39/39M [48] and ASTM C78/78M [48]. This study aims to provide data on both the mechanical properties of cob and the volume shrinkage values. The proposed manufacturing method is highly accessible, requiring neither machinery nor fibre pretreatment.

## 4. Conclusions

A study was conducted on the mechanical behaviour of cob samples as a function of age and of the volume shrinkage of these samples as a function of fibre content. The mixtures consisted of clay, water, and 3% or 6% wheat fibre. A control mixture was made with no fibre. Tests were conducted on three samples per mixture. Based on the results obtained after the samples were tested without fibre and with 3% and 6% fibre reinforcement and on the subsequent analysis, the following conclusions were drawn:

- An analysis of the drying kinetics showed that the addition of fibres to the clay mix reduces the volume shrinkage rate of clay materials while eliminating cracking, but the extent depends wholly on how the fibres are used (dry or wet).
- To eliminate surface cracking, the fibres must be wetted with a suitable quantity of water for 10 to 24 h before mixing. Moreover, the addition of the fibres reduces the density of the cob materials, thus reducing the weight of the cob structures.
- An analysis of the results of the mechanical strength of the samples showed that the reinforcement with wheat fibres improves the mechanical properties, although the samples were manually made with bulk fibres.
- The samples reinforced with wheat fibres have a plastic behaviour enabling walls constructed with these materials to withstand environmental stresses such as earthquakes and strong winds without collapsing.
- An ANOVA and *t*-test analysis revealed significant values, showing that at 28 days of drying, the samples without fibres exhibited higher flexural strength than those with fibres. However, at 120 days of drying, the flexural strength values of the samples with fibres improved to match those of the samples without fibres, indicating a progressive enhancement in the flexural strength of the fibre-reinforced samples over time.

As a general conclusion, cob exhibits a relatively ductile behaviour as compared to the brittle behaviour of earth materials without fibre reinforcement. This behaviour is strongly influenced by the presence of fibres in the samples. The capacity of a building to deform without collapsing is essential to saving human lives and repairing structures, and as a filler material for wooden structures, cob can enhance this capacity.

Based on the results presented and the work in progress within the project, further studies are required to complete this study:

- Experimental studies on cob specimens manufactured with different types of plant fibres need to be developed using the same manufacturing method.
- There should be a study of the influence of water content on the mechanical properties and volumetric shrinkage of cob to observe the influence of varying water content on the compressive and flexural strength of cob specimens manufactured using the traditional method.
- Modelling the volume shrinkage of the cob with 3% and 6% fibres as a function of time is important, using real climatic data to observe how cob reacts when the climate varies.
- Studying the behaviour of cob walls in the face of earthquakes and high winds (vertical/horizontal/diagonal wood-frame systems filled with cob) is also important. A study on the cost savings of this type of construction compared to conventional material construction would be necessary to confirm all the benefits associated with cob construction.

## Figures and Tables

**Table 1:** Mix proportions (grams).

Fibre Content (%) (Fibre/Clay)	Clay Mass	Water Mass (Water/Clay = 25%)	Mass of Wheat Fibre	Mass of Water for Wheat Humidification Water/Fibre = 205%
0	5000	1250	0	0
3	4850	1212.5	150	307.5
6	4700	1175	300	615

**Table 2:** Sample production steps.

Steps	Description	Step Duration
1	Fibre humidification	1 to 2 h
2	Mixture of clay and water	Approx. 15 min
3	Addition of fibre: mixing clay, water, and humidified fibres to obtain a homogenous mixture	Approx. 15 to 20 min
4	Total mixture rest time	2 to 3 h

**Table 3:** Mineralogical characteristics of red and beige clay: XRD analysis.

Mineralogical Compositions (Weight %)					
Type of Clay	Kaolinite	Quartz (Silicates)	Illite	Muscovite (Mica)	Hematite
Red	8.9	42.6	21.8	24.5	2.2
Beige	25.8	42.5	14.6	16.9	0.1

**Table 4:** Chemical characteristics of the red and beige clays: XRF analysis.

Chemical Compositions (Weight %)					
	H_2_O	MgO	Al_2_O_3_	SiO_2_	K_2_O
Red	2.4	0.8	19.9	70.1	4.7
Beige	4.4	0.5	21.3	70.4	3.2

## Data Availability

Data are contained within the article and Supplementary Materials.

## Supplementary Materials

The following supporting information was downloaded at https://www.mdpi.com/article/10.3390/ma17030736/s1, Figure S1. The condition of the samples after and before compression testing. Figure S2. Condition of the samples after and before flexural testing. Table S1. Influence fibres on compressive strength. Table S2. Effect of time on mechanical properties. Table S3. Influence fibres on flexural strength.
