# Supplementary material for: The Impact of Vegetable Fibres on the Shrinkage and Mechanical Properties of Cob Materials
Source: Materials (Basel). 2024 Feb 3;17(3):736. doi: 10.3390/ma17030736 (PMC10856543; doi:10.3390/ma17030736)
Supplement: Supplementary file 1 [file materials-17-00736-s001.zip › materials-2825239-supplementary-new version.pdf]

Article

The Impact of Vegetable Fibers on the Shrinkage and Mechanical Properties of Cob Materials

Aguerata Kabore * and Claudiane M. Ouellet-Plamondon

Department of Construction Engineering, Ecole de Technologie Supérieure, 1100 Notre-Dame Street, Montreal, QC H3C 1K3, Canada; claudiane.ouellet-plamondon@etsmtl.ca
* Correspondence: aguerata.kabore.1@ens.etsmtl.ca

SUPPLEMENTARY INFORMATION

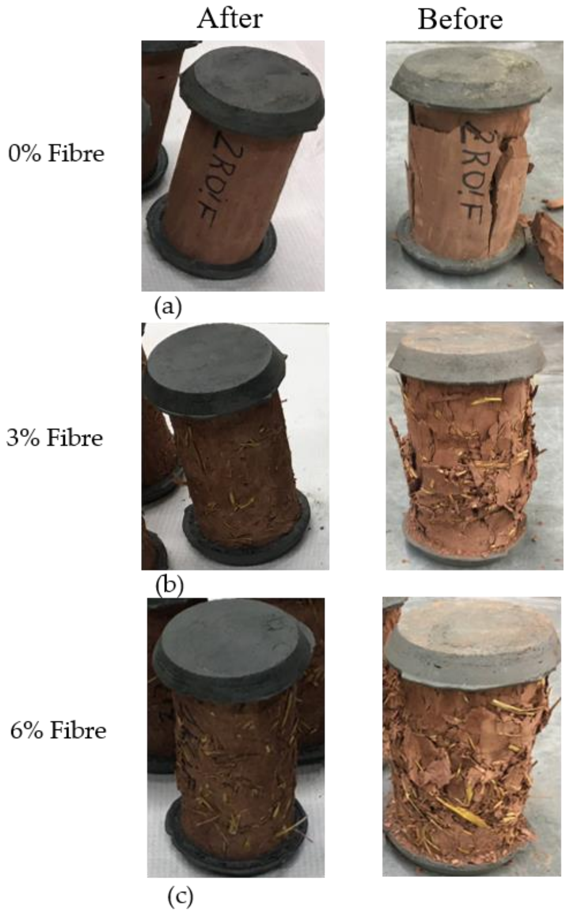

Figure S1. The condition of the samples after and before compression testing.

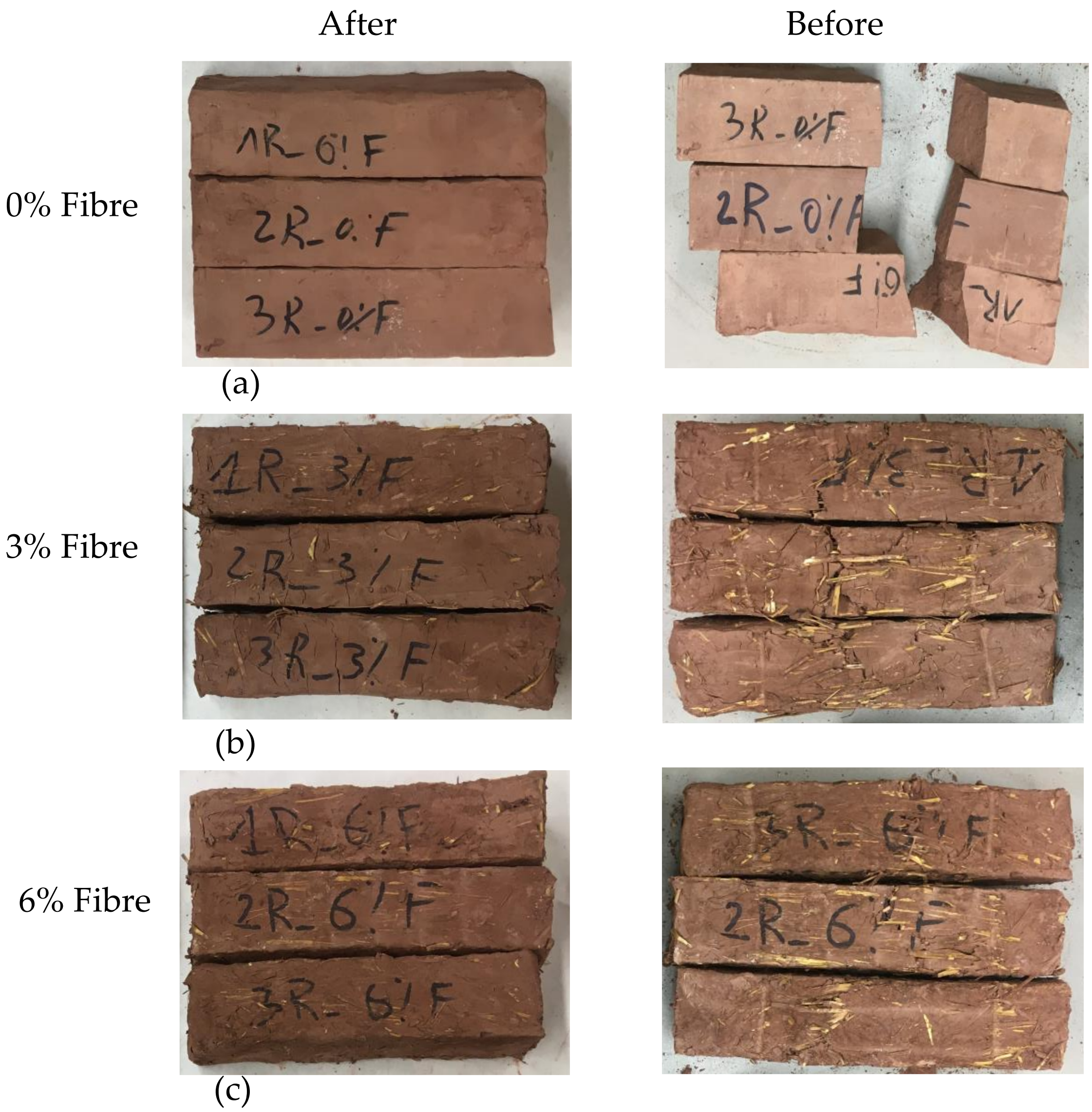

Figure S2. Condition of the samples after and before flexural testing.

ANOVA and t-TEST

Table S1. Influence fibres on compressive strength.

ANOVA - Significant difference observed.

Time (days)	Groups (fibre)	Count	Average	Stand. dev.	Variance	P-value
	0	6	1.72	0.09	0.06	**5.03E-11**
28	3	6	2.13	0.09	0.04	
	6	6	4.59	0.03	0.16	
	0	6	1.40	0.19	0.023	**8.61E-10**
120	3	6	2.15	0.17	0.058	
	6	6	3.91	0.19	0.182	

Table S2. Effect of time on mechanical properties.

t-TEST- Significant difference observed.

	Compressive strength		Flexural strength	
Clay	Red	Beige	Red	Beige
Mean	2.81	2.49	0.83	1.25
Variance	1.77	1.25	0.08	0.05
Observations	18	18	18	18
P-value	**0.008**		**8.62E-05**	

Table S3. Influence fibres on flexural strength.

ANOVA A- Significant difference observed.

Time (days)	Groups (fibre)	Count	Average	Stand. dev.	Variance	P-value
28	0	6	1.10	0.23	0.08	**0.009**
	3	6	0.71	0.21	0.06	
	6	6	0.67	0.10	0.02	

ANOVA B- Non-significant difference observed.

Time (days)	Groups (fibre)	Count	Average	Stand. dev.	Variance	P-value
120	0	6	1.16	0.17	0.06	**0.17**
	3	6	1.21	0.12	0.02	
	6	6	1.39	0.14	0.05	
